# Distinct osmoregulatory responses to sodium loading in patients with altered glycosaminoglycan structure: a randomized cross-over trial

**DOI:** 10.1186/s12967-021-02700-0

**Published:** 2021-01-20

**Authors:** Eliane F. E. Wenstedt, Jetta J. Oppelaar, Stijn Besseling, Nienke M. G. Rorije, Rik H. G. Olde Engberink, Arie Oosterhof, Toin H. van Kuppevelt, Bert-Jan H. van den Born, Jan Aten, Liffert Vogt

**Affiliations:** 1 Department of Internal Medicine, Section of Nephrology, Amsterdam Cardiovascular Sciences, Amsterdam UMC, University of Amsterdam, Meibergdreef 9, Amsterdam, The Netherlands; 2 Department of Biochemistry, Radboud UMC, Geert Grooteplein Zuid 10, Nijmegen, The Netherlands; 3 Department of Internal Medicine, Section of Vascular Medicine, Amsterdam Cardiovascular Sciences, Amsterdam UMC, University of Amsterdam, Meibergdreef 9, Amsterdam, The Netherlands; 4 Department of Pathology, Amsterdam UMC, University of Amsterdam, Meibergdreef 9, Amsterdam, The Netherlands; 5 Department of Internal Medicine, Section of Nephrology, Amsterdam UMC, Room D3-324, Meibergdreef 9, P.O. Box 22660, 1100 DD Amsterdam, The Netherlands

**Keywords:** Sodium, Glycosaminoglycan, Osmoregulation, Type 1 diabetes, Hereditary multiple exostoses, Nuclear factor of activated T-cells 5

## Abstract

**Background:**

By binding to negatively charged polysaccharides called glycosaminoglycans, sodium can be stored in the body—particularly in the skin—without concurrent water retention. Concordantly, individuals with changed glycosaminoglycan structure (e.g. type 1 diabetes (DM1) and hereditary multiple exostosis (HME) patients) may have altered sodium and water homeostasis.

**Methods:**

We investigated responses to acute (30-min infusion) and chronic (1-week diet) sodium loading in 8 DM1 patients and 7 HME patients in comparison to 12 healthy controls. Blood samples, urine samples, and skin biopsies were taken to investigate glycosaminoglycan sulfation patterns and both systemic and cellular osmoregulatory responses.

**Results:**

Hypertonic sodium infusion increased plasma sodium in all groups, but more in DM1 patients than in controls. High sodium diet increased expression of nuclear factor of activated t-cells 5 (NFAT5)—a transcription factor responsive to changes in osmolarity—and moderately sulfated heparan sulfate in skin of healthy controls. In HME patients, skin dermatan sulfate, rather than heparan sulfate, increased in response to high sodium diet, while in DM1 patients, no changes were observed.

**Conclusion:**

DM1 and HME patients show distinct osmoregulatory responses to sodium loading when comparing to controls with indications for reduced sodium storage capacity in DM1 patients, suggesting that intact glycosaminoglycan biosynthesis is important in sodium and water homeostasis.

*Trial registration* These trials were registered with the Netherlands trial register with registration numbers: NTR4095 (https://www.trialregister.nl/trial/3933 at 2013-07-29) and NTR4788 (https://www.trialregister.nl/trial/4645 at 2014-09-12).

## Background

Disturbances in osmoregulation result in abnormal plasma sodium concentrations leading to either hypo- or hypernatremia; the most common electrolyte disorders in clinical practice [1]. Dysnatremia is to be found in one-third of elderly patients admitted to the emergency room and one-third of critically ill patients admitted to the intensive care unit [2, 3]. Dysnatremias have a large impact on both short- and long-term outcomes, as illustrated by many studies that demonstrate an association between dysnatremia and increased morbidity and mortality [2, 4–6]. Yet, the pathophysiology of disturbed osmoregulation remains poorly understood and complete understanding of water and sodium homeostasis is thought to be necessary for the improvement of outcomes [7].

In recent years, the classical view on sodium homeostasis has drastically changed by the revelation that sodium can be transiently stored within various tissues of the body in concentrations that far exceed those in plasma, including skin, muscle, and possibly the blood vessel wall, varying with dietary intake [8–10]. This adds a third dynamic compartment to the classical two-compartment model, which solely assumes equal osmolality in the intra- and extracellular space. Recently, the potential sodium buffering capacity in healthy subjects was estimated to be ~ 50 mmol (equaling ~ 3 g of salt (NaCl)) following acute hypertonic infusion [11]. Sodium storage is believed to be facilitated by negatively charged polymeric disaccharides called glycosaminoglycans (GAGs) [12, 13]. GAGs can be variously sulfated, and the sulfation degree may primarily determine the quantity of GAG-mediated tissue sodium accumulation [14–16]. Several studies in animal models and in healthy humans have shown that increased skin sodium storage (induced by high sodium intake) coincides with increases in GAGs and enzymes involved in the modification and degradation of GAGs [10, 14–18]. Also, nuclear factor of activated T-cells 5 (NFAT5; also called tonicity-responsive enhancer binding protein (TonEBP)), a transcription factor that is crucial for tissue specific cellular responses to hypertonic stress, responds to high tissue sodium concentrations [10, 14–18]. Thus, individuals with changes in GAG biosynthesis and/or structure may have altered sodium homeostasis and respond differently to sodium overloads. Whether patients with disturbed GAG metabolism are characterized by disturbed osmoregulatory responses to sodium is unknown.

We studied the osmoregulatory responses to acute and chronic sodium loading in type 1 diabetes mellitus (DM1) patients, known for acquired GAG remodeling, and patients with hereditary multiple exostosis (HME) with genetic alterations in GAG synthesis, and compared them to matched healthy controls. We assessed systemic osmoregulatory responses by measurements of plasma and urine osmolarity, and local cellular osmoregulatory responses by assessing NFAT5 (along with associated GAG changes) in skin biopsies. In DM1, a decrease in GAG synthesis and/or increase in GAG destruction results in decreased GAG content and GAG sulfation degree in a variety of organs and structures, including the kidney, skin and endothelial surface layer (luminal coating of blood vessel walls) [19–22]. Patients with HME, a rare autosomal dominant disorder with mutations in EXT-1 and/or EXT-2 genes that are responsible for polymerization of heparan sulfate (specific type of GAG), have a genetic form of GAG dysfunction [23]. Data from recent animal experiments using congenic EXT-1^±^EXT-2^±^ mice show a damaged endothelial surface layer and lower skin sodium-to-glycosaminoglycan ratio [24, 25].

## Methods

### Study design

We carried out two similar prospective randomized cross-over intervention studies in DM1 patients, HME patients, and healthy controls. We included male, non-smoking, normotensive (Blood pressure (BP) < 140/90 mmHg) subjects between 18 and 40 years old with a body mass index (BMI) < 30 kg/m^2^. For inclusion, DM1 patients had to have normal and stable renal function (creatinine clearance > 60 ml/min and < 6 ml/min decline per year) and HbA1c (42–86 mmol/mol) during 6 months preceding the study. Use of renin-angiotensin system blocking agents was allowed for DM1 patients, but these were discontinued prior to the study visits using an interval that was equal to or exceeded 5 times the elimination half-life. All subjects pursued an 8-day low sodium diet (LSD; < 1.2 g (50 mmol) sodium/day) and high sodium diet (HSD > 4.8 g (200 mmol) sodium/day) in a randomized order, with 1–2 weeks between diets. Diet order was determined by block randomization via sealed envelopes by the study investigators. Diet status was not masked for study subjects or investigators during the dietary intervention. Diets were pursued with the help of a dietary list, based on which participants could compile their own diet. This list advised to resemble the normal diet of the individual as much as possible—e.g., by adding extra salt instead of changing the whole dietary pattern (aiming to keep other nutrients as stable as possible). We checked dietary compliance by collecting 24-h urine samples on day 3, 6 and 8. After each diet, skin biopsies were obtained.

### Acute sodium loading experiment

On day 8 of LSD, an additional 1-day acute sodium loading experiment was performed in all subjects. After standardized low-sodium breakfast and lunch, subjects were asked to fully empty their bladder, and hypertonic sodium was administered intravenously in 30 min. In each subject, the total amount of infused sodium equaled 5 mmol/L of total body water (estimated to be 60% of total body weight). By adding a 20% NaCl solution (range 29–57 mL in DM1, 35–55 mL in HME, and 29–50 mL in healthy controls (HC)) to 500 mL of 0.9% NaCl, the infused volume was only slightly different. After infusion, we obtained blood and urine samples at timed intervals during a 4-h period. During this period, water intake was standardized to a total of 350 mL divided in similar portions on similar time points in all subjects. DM1 patients took their standard dose of insulin during the day. The results of the acute sodium loading experiment in healthy controls and data on urinary GAGs were previously published [11, 26].

### Calculations acute sodium loading experiment

To assess whether there were indications for (differences in) sodium buffering after acute sodium loading in DM1 patients and HME patients, we compared observed plasma and urinary cation levels with expected plasma and urinary cation levels according to the Adrogue-Madias and the Nguyen-Kurtz formulas (Additional file 1: Appendix 1), as described previously [11, 27]. In short, with these formulas, we calculated expected plasma sodium levels based on the administered sodium and water quantity, taking into account total body water and initial plasma sodium [28, 29]. Furthermore, we calculated how much cations should be present in urine to account for plasma sodium decreases that took place after initial increases. The discrepancy between observed and expected values indicates which fraction of infused sodium—provided that plasma potassium concentrations remain stable—is stored in the body. In DM1 patients, plasma sodium levels were corrected for glucose levels, using a correction factor of 1.6 mmol/L per glucose increase of 5.5 mmol/L [30].

### GAG analysis in 24-h urine collection

Glycosaminoglycans were enzymatically digested into disaccharides, and results were reported as previously described [31, 32]. Disaccharides D0a4 and D0a10 were formed after enzymatic digestion with chondroitinase B representing dermatan sulfate disaccharides, and with chondroitinase C representing chondroitin sulfate disaccharides. Disaccharide concentrations were adjusted for creatinine concentration of the urine samples. A validated high performance liquid chromatography with mass spectrometry/mass spectrometry method was used to quantify urinary excretion of heparan sulfate, dermatan sulfate, and chondroitin sulfate disaccharides. Values below the lower limit of quantification were assigned as of the numeric half value of the lower limit of quantification.

### Other laboratory analyses

Blood was collected in 4.5-ml lithium heparin tubes (BD Vacutainer, Becton Dickinson, Franklin Lakes, NJ) for analysis of plasma sodium, potassium, osmolality, and creatinine. Three-milliliter tubes with 5.4-mg spray-dried K2 ethylenediaminetetraacetic acid (BD Vacutainer) were used for hematocrit determination. All blood samples were centrifuged at 2000 g for 10 min at 18 ℃ and analyzed within 60 min of collection. We used the indirect ion selective electrode method to measure plasma sodium and potassium and urine sodium and potassium. Plasma and urinary osmolality were determined by freezing point depression.

### Immunohistochemistry

To assess cellular osmoregulation responses to sodium, sections of formalin-fixed and paraffin-embedded skin biopsies were investigated for presence of NFAT5 and glycosaminoglycans by histochemistry and immunostaining as described in detail in Additional file 1: Appendix 2. Briefly, sections were incubated with rabbit IgG anti-NFAT5 (ThermoFisher), followed by AlexaFluor488-conjugated goat IgG anti-rabbit IgG (Jackson ImmunoResearch). NFAT5-stained sections were counterstained using Hoechst 33,342 nuclear stain (ThermoFisher). For detection of total GAGs, tissue sections were stained with Alcian Blue 8GX (Sigma-Aldrich, Saint Louis, MO, USA) at pH 2.7 and at pH 1.0, at which strongly acidic sulfated glycosaminoglycans are stained more selectively. Sections were counterstained with Nuclear Fast Red (Sigma-Aldrich, Saint Louis, MO, USA). For staining of dermatan sulfate GAGs, VSV-tagged phage display derived antibody LKN1 (Radboudumc, Nijmegen, the Netherlands) was applied to detect 4/2,4-di-O-sulfated dermatan sulfate domain [33] and GD3A12 antibody (Radboudumc, Nijmegen, the Netherlands) to detect IdoA-Gal-NAc4S dermatan sulfate domain [33, 34]. For heparan sulfate GAGs, VSV-tagged phage display derived antibodies (HS4C3, AO4B08 and HS4E4) (Radboudumc, Nijmegen, the Netherlands) were applied as primary probes to detect specific sulfation motifs in heparan sulfate chains. In particular, HS4C3 binds 3-O-sulfated heparan sulfate chains with preference for the fully sulfated IdoA2S0GlcNS3S6S domains [35], AO4B08 binds N-sulfated, 2-O-sulfated, and 6-O-sulfated heparan sulfate chains, corresponding to the IdoA2S-GlcNS6S domain which lacks 3-O sulfatations [36] and HS4E4 binds heparan sulfate domains containing both N-sulfation and N-acetylation and in general heparan sulfate chains with low sulfation grades since the presence of 6-O-sulfated sites inhibits binding of HS4E4 [36]. Regarding the spectrum of these three domains of heparan sulfate, we therefore consider binding of HS4C3 to represent a highly sulfated heparan sulfate domain, AO4B08 a moderately sulfated heparan sulfate domain and HS4E4 a low sulfated heparan sulfate domain. Bound antibodies were detected using subsequently rabbit IgG anti-VSV (Sigma), poly-AP conjugated goat IgG anti-rabbit IgG (Bright Vision, Immunologic) and PermaBlue/AP substrate (Diagnostic Biosystems). The sections were counterstained with Fluorescin Ulex Europaeus Agglutin (Vector Laboratories) which was detected by Ulex Europaeus Lectin Type I Rabbit anti Ulex (Dako) and BrightVision Poly-HRP anti-rabbit IgG (ImmunoLogic). HRP activity was visualized using NovaRED (Vector Laboratories).

### Image analysis

Epifluorescence imaging of NFAT5-stained sections was performed (Leica DM5500B, Wetzlar, Germany) with an HCX PL APO 63×/1.40–0.60 oil immersion objective and applying filters A4 for Hoechst and L5 for AlexaFluor488 fluorochromes. From each section six to ten images were recorded of the epidermis and conjointly of the dermis using a DFC365 FX camera and LASX software (Leica). For manually selected regions of interest, background was substracted using the ‘rolling ball’ plugin with ImageJ2 software [37]. The number of nuclei was analyzed for each image. Next, the fractional AlexaFluor488-stained surface area (representing NFAT5) was measured and expressed per nucleus for at least 100 nuclei per region of interest. Brigthfield microscopy was applied for sections stained with Alcian Blue, LKN1, GD3A12, HS4C3, AO4B08 and HS4E4 using either a BX51 with UPlanXAPO 20x/0.80 objective and DP70 camera (Olympus, Tokyo, Japan) or a slide scanner (IntelliSite Ultra Fast Scanner; Philips, Eindhoven, the Netherlands) with UPlanXAPO 40×/0.95 objective (Olympus). In recorded images either dermis or epidermis were selected as region of interest. Subsequently, the ‘color deconvolution’ plugin with setting for FastBlue/FastRed/DAB was applied to isolate the Alcian Blue and FastRed or the PermaBlue and NovaRed signals [38]. The thresholds were set with the AutoThreshold function “Moments”. Finally, the fractional stained area of the regions of interest was measured for PermaBlue stained sections.

Furthermore, to validate the semi-quantitative measurements and to specify the measurements on the cellular level, images from all stainings were analyzed qualitatively by two blinded reviewers, according to a pre-determined scoring system. The extent of staining was scored for the dermal extracellular papillary matrix, papillary cells and endothelium. For the diet type, the researchers remained blinded during all staining and analysis processes.

### Statistics

Continuous data are expressed as mean and standard error of the mean (SEM). Multiple imputation was used to estimate missing values, and potential outliers were detected using Grubb’s test. Paired t-test or Wilcoxon test was used to detect differences between LSD and HSD, depending on the data distribution. To determine differences between patient groups an unpaired t-test or Kolmogorov–Smirnov was performed, as appropriate. Test hypotheses were (i) H_0_: DM1 patients = healthy controls, H_A_: DM1 patients ≠ healthy controls and (ii) H_0_: HME patients = healthy controls, H_A_: HME patients ≠ healthy controls. To test correlations between variables Spearman’s correlation coefficient was used. For the acute hypertonic saline infusion experiment, repeated measurements two-way ANOVA with Tukey post-hoc test was used to account for repeated time points. The statistical analysis was executed using SPSS software (version 24.0, IBM Analytics, Armonk, NY, USA). Figures were acquired using GraphPad Prism (version 7.0d, GraphPad, La Jolla, CA, USA). A P-value of < 0.05 was considered significant.

## Results

### Population and dietary intervention

**Fig. 1:**
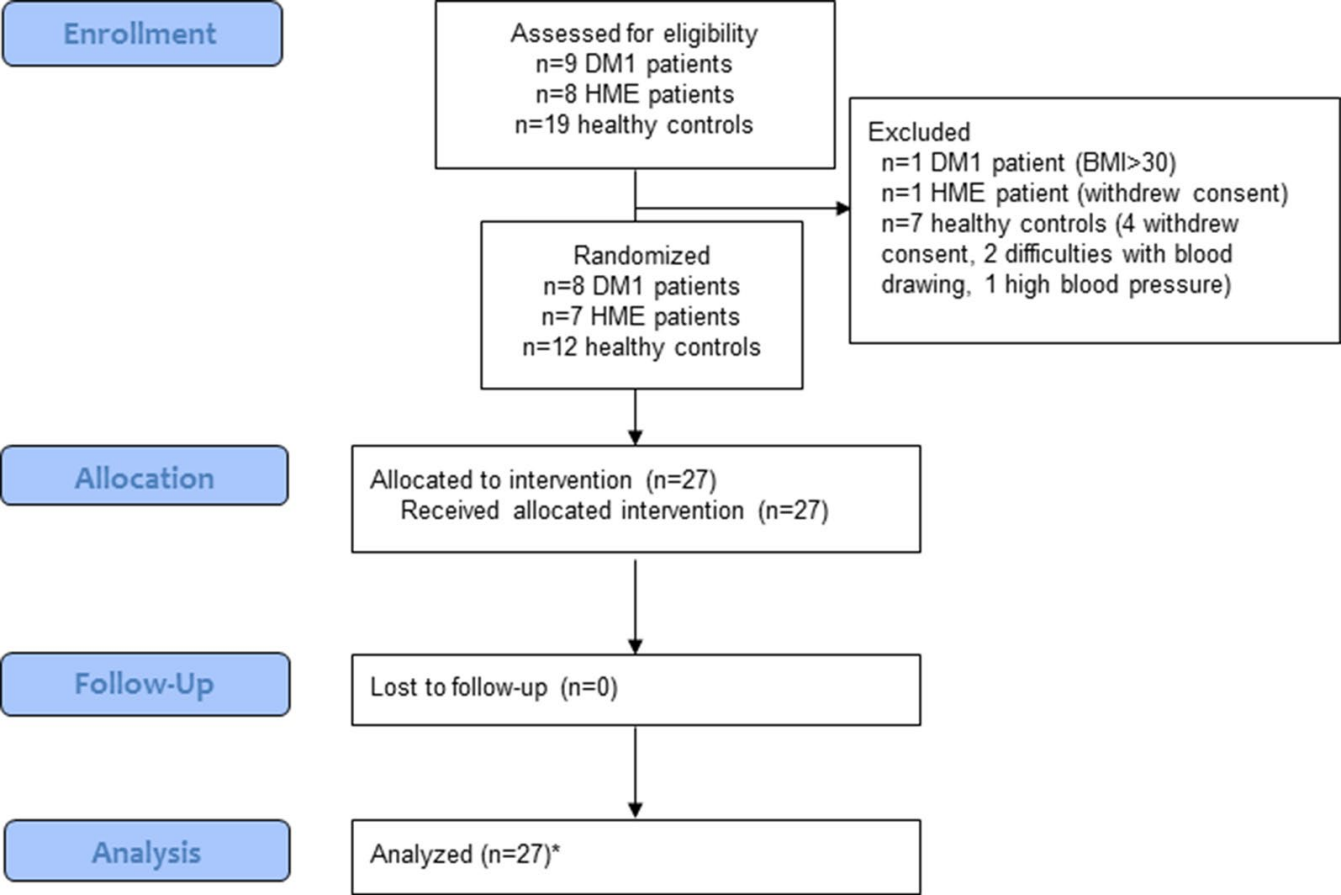
Flow chart of inclusion. All 27 participants (8 DM1 patients, 7 HME patients, and 12 healthy controls) were allocated to and received the allocated intervention of LSD, HSD, and acute hypertonic saline infusion. *for the acute hypertonic saline infusion experiment, 11 healthy controls were analyzed, since blood sampling within the first 10 min after infusion proved to be problematic in one healthy control. DM1, type 1 diabetes. HME, hereditary multiple exostoses. LSD, low salt diet. HSD, high salt diet

Between September 2013 and November 2015 we screened 9 DM1 patients, 8 HME patients, and 19 healthy controls. One of the DM1 patients was excluded after screening because of a BMI > 30 kg/m^2^ and one HME patient withdrew informed consent after screening due to inability to adhere to the protocol instructions. Of the healthy controls, four subjects withdrew their consent after inclusion before randomization and three subjects were excluded before randomization (one due to high BP and two others due to difficulties with blood drawing). There was no loss-to-follow-up and all randomized subjects were included in our analyses (Fig. 1). Baseline characteristics are depicted in Table 1.

**Table 1:** Baseline characteristics of study subjects, determined at screening before commencement of the diets

	Type 1 diabetes patients (n = 8)	HME patients (n = 7)	Healthy controls (n = 11)
Age (year)	28.1 (2.0)*	26.6 (3.2)	22.7 (1.2)
BMI (kg/m^2^)	22.8 (0.9)	24.4 (1.1)	22.0 (0.6)
Total body weight (kg)	77.4 (3.3)	78.8 (2.8)	75.7 (2.0)
Hematocrit (L/L)	0.45 (0.01)*	0.44 (0.01)	0.43 (0.01)
Glucose (mmol/L)	8.1 (1.5)*	4.7 (0.2)	5.1 (0.2)
Plasma sodium (mmol/L)	139.0 (0.5)	140.1 (0.3)	140.0 (0.5)
Plasma potassium (mmol/L)	4.4 (0.1)	4.0 (0.1)	4.2 (0.1)
Plasma osmolality (mOsm/kg)	295 (2)	290 (1)	287 (5)
Plasma creatinine (umol/L)	72 (3)*	73 (2)*	81 (3)
Urine sodium (mmol/24 h)	193 (28)	154 (24)	166 (20)
Urine potassium (mmol/24 h)	74 (14)	72 (4)	71 (5)
Supine systolic blood pressure (mmHg)	124 (2)	118 (2)	121 (3)
Supine diastolic blood pressure (mmHg)	64 (3)	63 (2)	59 (2)
Supine heart rate (bpm)	56 (2)	64 (4)	61 (2)

Data are depicted as mean (SEM). Data were tested using an unpaired t-test compared to controls. *P < 0.05 vs. healthy controls

All subjects adequately followed the LSD and HSD, as assessed by 24-h urine sodium excretion. Urinary sodium excretion was comparable among groups, equaling a mean of ~ 1.5 g estimated NaCl intake per day for LSD and a mean of ~ 19 g estimated NaCl intake per day for HSD (Table 2). Potassium intake was successfully kept stable (Table 2).

**Table 2:** Characteristics of the study subjects after LSD and HSD

	Type 1 diabetes patients (n = 8)		HME patients (n = 7)		Healthy controls (n = 11)	
	LSD	HSD	LSD	HSD	LSD	HSD
Body weight (kg)	75.6 (3.1)	78.2 (3.4)*	77.1 (3.2)	79.5 (2.9)*	74.0 (1.9)	76.5 (1.9)*
Plasma sodium (mmol/L)	137.3 (0.8)	139.8 (0.8)*	139.3 (0.7)	140.1 (0.3)	137.5 (0.5)	140.3 (0.5)*
Plasma potassium (mmol/L)	4.3 (0.1)	4.2 (0.1)	3.8 (0.1)	3.9 (0.1)	3.9 (0.1)	3.9 (0.1)
Plasma osmolality (mOsm/kg)	290.3 (0.7)	297.6 (2.1)*	286.0 (0.8)	289.6 (0.9)*	284.9 (0.9)	289.6 (1.1)*
Plasma glucose (mmol/L)	10.7 (1.4)	11.0 (1.0)	5.2 (0.2)	5.3 (0.2)	4.9 (0.1)	5.0 (0.1)
Urine sodium (mmol/24 h)	23.3 (4.6)	352.7 (25.7)*	23.8 (4.4)	291.9 (25.3)*	19.1 (2.8)	340.1 (30.0)*
Urine potassium (mmol/24 h)	113.8 (12.9)	105.8 (13.8)	69.5 (15.2)	87.3 (10.1)	87.7 (7.5)	89.6 (5.9)
Fractional sodium excretion (%)	0.08 (0.01)	1.0 (0.1)*	0.1 (0.01)	1.0 (0.1)*	0.1 (0.01)	1.1 (0.1)*
Free water clearance (L/24 h)	−0.9 (0.6)	−3.0 (0.6)*	−0.1 (0.3)	−2.0 (0.2)*	−0.7 (0.2)	−2.8 (0.1)*
Electrolyte-free water clearance (L/24 h)	1.3 (0.3)	−0.5 (0.3)*	1.2 (0.2)	−0.7 (0.3)*	0.9 (0.2)	−1.2 (0.1)*

Data are depicted as mean (SEM). Data were tested using a paired t-test (LSD vs. HSD). LSD, low sodium diet. HSD, high sodium diet. *P < 0.05 vs. LSD

### Urinary GAGs at baseline

**Fig. 2:**
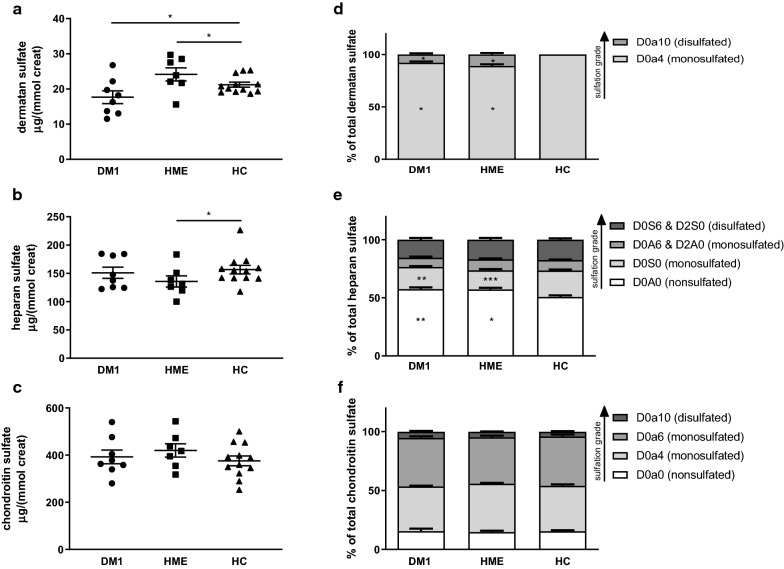
Total amount and sulfation grades of GAGs in 24-h urine after LSD. (**a-c**) DM1 patients have less urinary dermatan sulfate than healthy controls. HME patients have less urinary heparan sulfate, but more urinary dermatan sulfate than healthy controls. **d** In healthy controls, all the dermatan sulfate consisted of D0a4 disaccharides, while in DM1 patients and HME patients also D0a10 disaccharides were found. The notation is as follows [32]: D0a4 = ΔUA-GalNAc4S, D0a10 = ΔUA-GalNAc4S6S. **e** There were significantly more nonsulfated disaccharides (D0A0) of heparan sulfate in DM1 patients and HME patients than in healthy controls. The notation is as follows [32]: D0A0 = ΔUA-GlcNAc, D0S0 = ΔUA-GlcNS, D0A6 = ΔUA-GlcNAc6S, D2A0 = ΔUA2S-GlcNAc, D0S6 = ΔUA-GlcNS6S, D2S0 = ΔUA2S-GlcNS. **f** The percentages disaccharides of chondroitin sulfate were equal among the three groups. The notation is as follows [32]: D0a0 = ΔUA-GalNAc, D0a4 = ΔUA-GalNAc4S, D0a6 = ΔUAGalNAc6S, D0a10 = ΔUA-GalNAc4S6S. DM1, type 1 diabetes. HC, healthy controls. HME, hereditary multiple exostosis. *n* = 8 (DM1), *n* = 7 (HME), *n* = 12 (HC). *P < 0.05 vs. healthy controls. **P < 0.01 vs. healthy controls. ***P < 0.001 vs. healthy controls. Data are presented as individual data with mean (SEM). Data were tested with an unpaired t-test or Kolmogorov–Smirnov test, as appropriate

On day 8 of LSD,urinary GAGs were measured to assess overall GAG status. DM1 patients had less urinary dermatan sulfate compared to healthy controls. Although urinary levels of heparan sulfate were similar for DM1 patients and healthy controls, the extent of sulfation of heparan sulfate was diminished in DM1 patients (Fig. 2). HME patients had lower along with less-sulfated urinary heparan sulfate than healthy controls, but, in contrast, had more urinary dermatan sulfate (Fig. 2). In both DM1 patients and HME patients, the di-sulfated dermatan sulfate disaccharide D0a10 was detected, contrasting the results found in healthy controls, where only mono-sulfated dermatan sulfate disaccharide was present.

### Acute sodium loading experiment

**Fig. 3:**
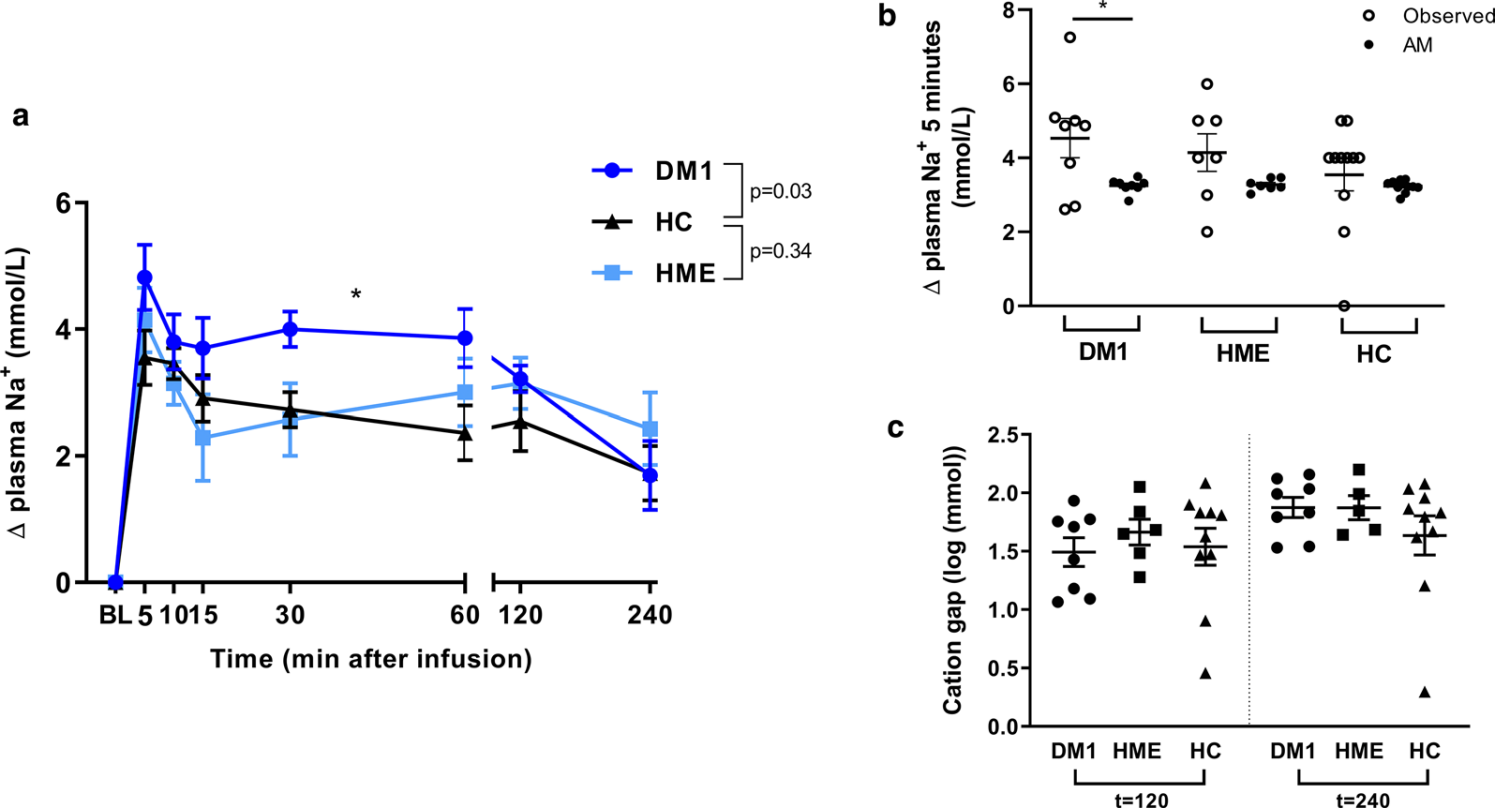
**a** Sodium homeostasis after acute hypertonic saline infusion. Absolute changes of plasma sodium compared to baseline (just before the start of infusion). Plasma sodium changes in DM1 patients were significantly different compared to healthy controls (patient group * time: p = 0.03). There was no difference between HME patients and healthy controls (patient group * time: p = 0.34). Data are presented as mean (SEM) and tested with a repeated measurements two-way ANOVA. **b** Differences between observed and expected plasma sodium changes 5 min after infusion. The difference between observed plasma sodium changes (5 min after infusion compared to baseline, i.e., just before the start of infusion) and expected plasma sodium changes as calculated with the Adrogue-Madias formula and Nguyen-Kurtz formula. In DM1 patients, the observed plasma sodium increase was higher than expected according to the formulas. In HME patients and healthy controls there was no discrepancy. For clarity, only the expected values of the Adrogue-Madias formula are depicted in the graph, comparison to the values of the Nguyen-Kurtz formula did not materially alter the results. Data are presented as mean (SEM) and tested with a Wilcoxon test. **c** The cation gap in different groups. The cation gap represents the cumulative difference between observed and expected cation levels in the urine, which reflects the amount of cations (and in case of stable potassium levels, likely the amount of sodium) that is “missing”, and is probably stored within the body. Data are presented as mean (SEM) and tested with an unpaired t-test (vs. healthy controls) after log transformation. DM1, type 1 diabetes. HME, hereditary multiple exostoses. HC, healthy controls. AM, Adrogue-Madias formula. *n* = 8 (DM1), *n* = 7 (HME), *n* = 12 (HC). *P < 0.05

To assess osmoregulation, plasma sodium changes subsequent to acute hypertonic saline infusion were measured (Fig. 3). Five minutes succeeding infusion, there was a plasma sodium increase compared to baseline in all three groups, after which plasma sodium decreased again, ending ~ 2 mmol above baseline 4 h after infusion. Plasma sodium changes in DM1 patients differed significantly compared to healthy controls (P = 0.03) with higher mean increases in DM1 patients up to 1 h after infusion (Fig. 3a). Additionally, in DM1 patients, the initial 5-min rise of plasma sodium was higher than expected according to the Edelman-based Adrogue-Madias and Nguyen-Kurtz formulas, whereas in HME patiens and healthy controls no discrepancies were found. (Fig. 3b). Changes in other laboratory parameters are depicted in Additional file 1: Figure S1 and Table S1. The amount of cations assumed to be in urine to account for plasma sodium decreases taking place between 5 min, 2 h and 4 h after infusion, was calculated using the Adrogue-Madias and Nguyen-Kurtz formulas. These amounts were substantially higher than the amounts truly observed in all groups (Fig. 3c and Additional file 1: Figure S2). This difference between observed and expected values represents the fraction of infused sodium and potassium ions that could not be traced back to plasma or urine (i.e., “cation gap”; and in case of stable plasma potassium most likely representing sodium ions i.e., “sodium gap”) and did not significantly differ compared to controls.

### Chronic sodium loading experiment

**Fig. 4:**
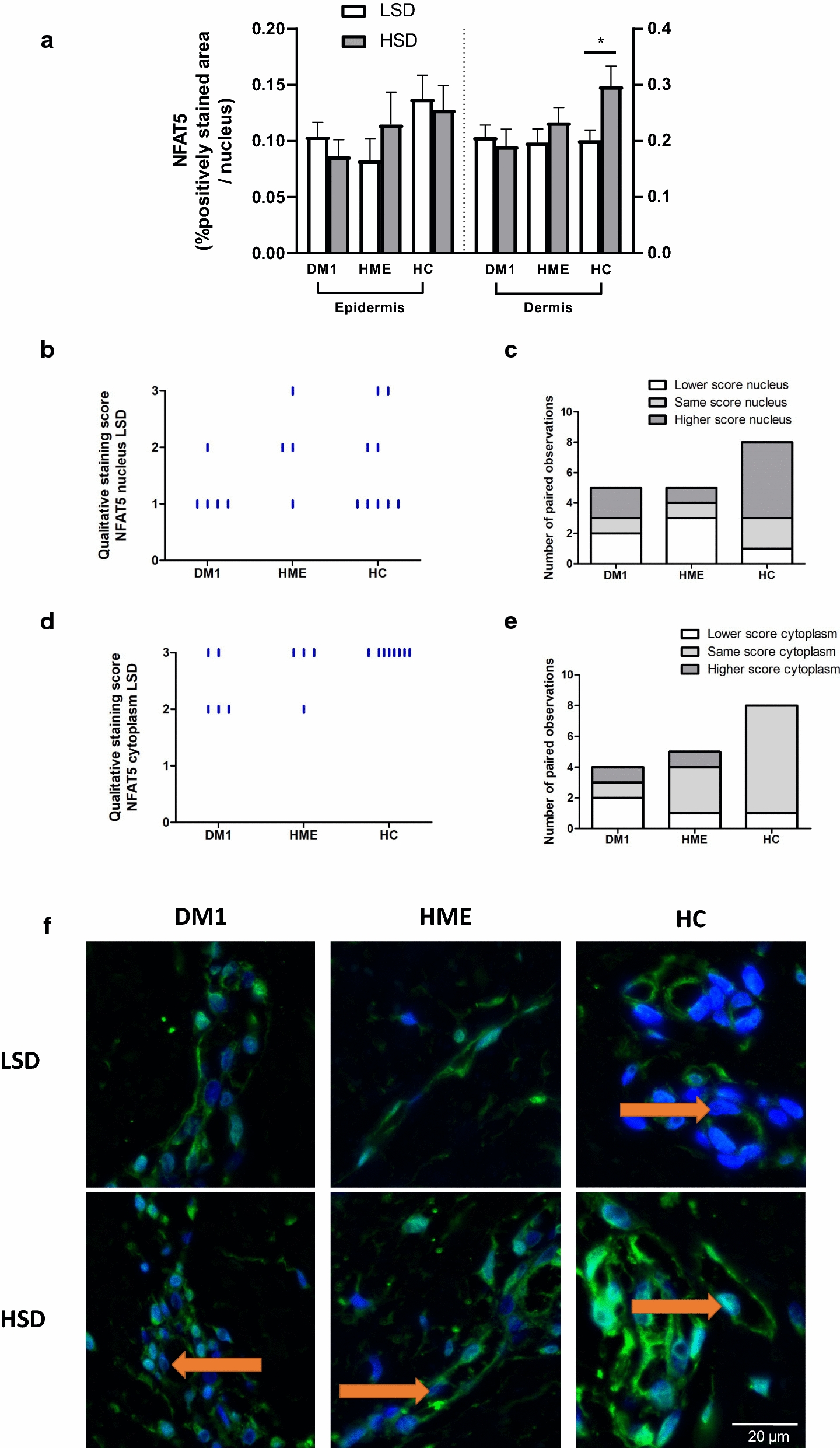
Expression of NFAT5 in the skin. **a** Semi-quantitative analysis. **b**, **d** Upon LSD no specific differences in NFAT5 expression could be observed in the endothelium. **c**, **e** Paired observations showed that in healthy controls the location of NFAT5 in endothelial cells shifted from a predominantly cytoplasmic pattern in LSD to a both cytoplasmic and nuclear pattern in HSD. No such shifts could be observed in other groups. **f** Paired histological images (of which a selection containing the endothelium is shown) show that in healthy controls the expression of NFAT5 (green) in endothelial cells shifts from a predominantly cytoplasmic pattern in LSD (nuclei visualized by Hoechst staining (blue)) to a cytoplasmic plus nuclear pattern in HSD. In DM1 and HME no shift could be observed. NFAT5, nuclear factor of activated T-cells 5. LSD, low sodium diet. HSD, high sodium diet. DM1, type 1 diabetes. HME, hereditary multiple exostosis. HC, healthy controls. *n* = 8 (DM1), *n* = 7 (HME), *n* = 12 (HC). *P < 0.05. Data are presented as individual data with mean (SEM). Data were tested with a paired t-test or Wilcoxon test, as appropriate, to compare two diets, and with an unpaired t-test or Kolmogorov–Smirnov test, as appropriate, to compare two patient groups

Skin NFAT5 and the presence of glycosaminoglycans were analyzed with immunohistochemistry in all groups. In healthy controls, HSD increased the extent of dermal NFAT5 expression (Fig. 4, Table 3). In other groups, no differences in dermal NFAT5 expression could be observed between both diets. The location of NFAT5 in endothelial cells shifted from a predominantly cytoplasmic pattern in LSD to a both cytoplasmic and nuclear pattern in HSD in healthy controls (Fig. 4b–f). No such HSD-induced nuclear translocation of NFAT5 could be observed in other groups. Alcian Blue staining at pH 2.7, representing total skin GAG, was similar in the papillary matrix in all groups after LSD (Table 3) (Additional file 1: Figure S3 and S4). There were salt-induced fluctuations in highly sulfated residues (after staining at pH 1.0) in healthy controls and HME patients. However, these fluctuations were not present in patients with DM1, showing a stable extent of highly sulfated residues upon HSD (Table 3) (Additional file 1: Figure S3). An increase or similar presence of total GAG content was present in DM1 patients, whereas in healthy controls a decrease was observed in the majority (Table 3) Additional file 1: Figure S4).

**Table 3:**
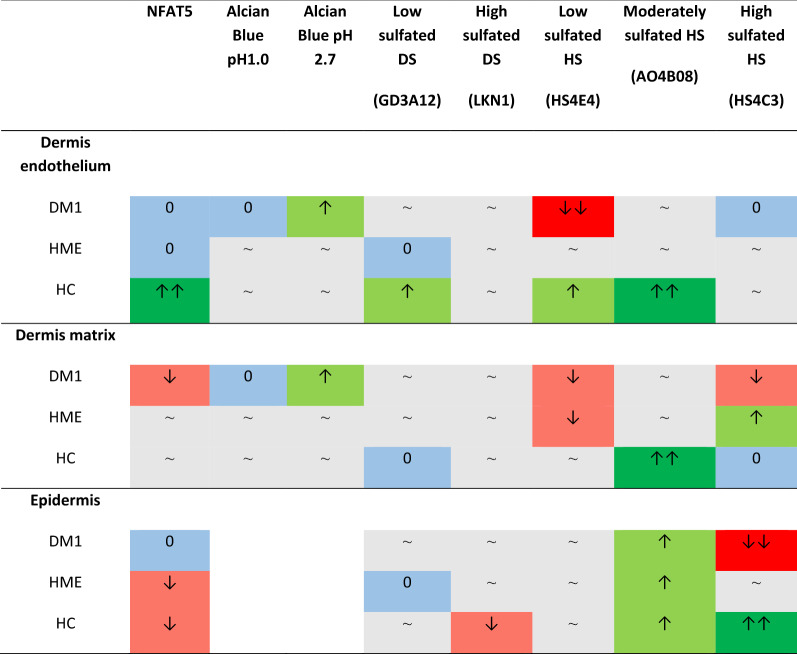
Overall results of paired observations of qualitative analysis

Trends of changes in staining extent are shown as ↑↑ (strong increase), ↑ (increase), ↓ (decrease), ↓↓ (strong decrease), 0 (same extent of staining), ~ non-specific fluctuations. Alcian Blue staining of the epidermis was not scored, due to minimal differences in extent of staining that could not be visually assessed. DM1, type 1 diabetes. HC, healthy controls. HME, hereditary multiple exostosis

**Fig. 5:**
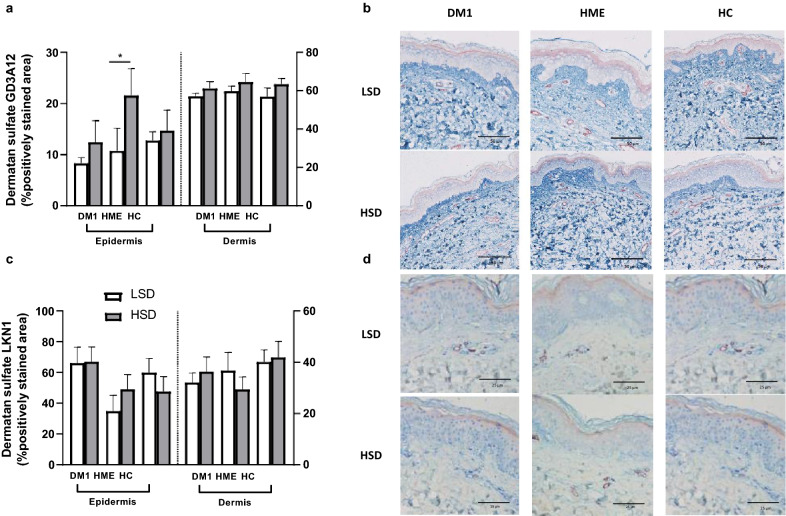
Expression of dermatan sulfate in epidermal and dermal skin. **a**, **c** Semi-quantitative analysis. **b** In HME patients an increase was measured in the extent of GD3A12 (blue) expression in the epidermis after HSD. **d** No diet-induced changes in expression of LKN1 (blue) could be observed in all groups. Blue represents GD3A12 or LKN1, respectively. Purple represents Ulex Europaeus Agglutinin I (UEA I). LSD, low sodium diet. HSD, high sodium diet. DM1, type 1 diabetes. HME, hereditary multiple exostosis. HC, healthy controls. *n* = 8 (DM1), *n* = 7 (HME), *n* = 12 (HC). *P < 0.05. Data are presented as individual data with mean (SEM). Data were tested with a paired t-test or Wilcoxon test, as appropriate, to compare two diets, and with an unpaired t-test or Kolmogorov–Smirnov test, as appropriate, to compare two patient groups

In the skin of HME patients, we semi-quantitatively measured an increase of the IdoA-Gal-Nac4S domain (GD3A12) in the epidermis after HSD (Fig. 5a, b). With qualitative analysis, we were unable to observe a cell-specific increase. Epidermal presence of the 4/2,4-di-O-sulfated (LKN1) domain of dermatan sulfate was not affected by HSD (Fig. 5c, d). In the dermis, we were unable to observe clear salt-induced differences in dermal expression of both domains of dermatan sulfate in all groups.

**Fig. 6:**
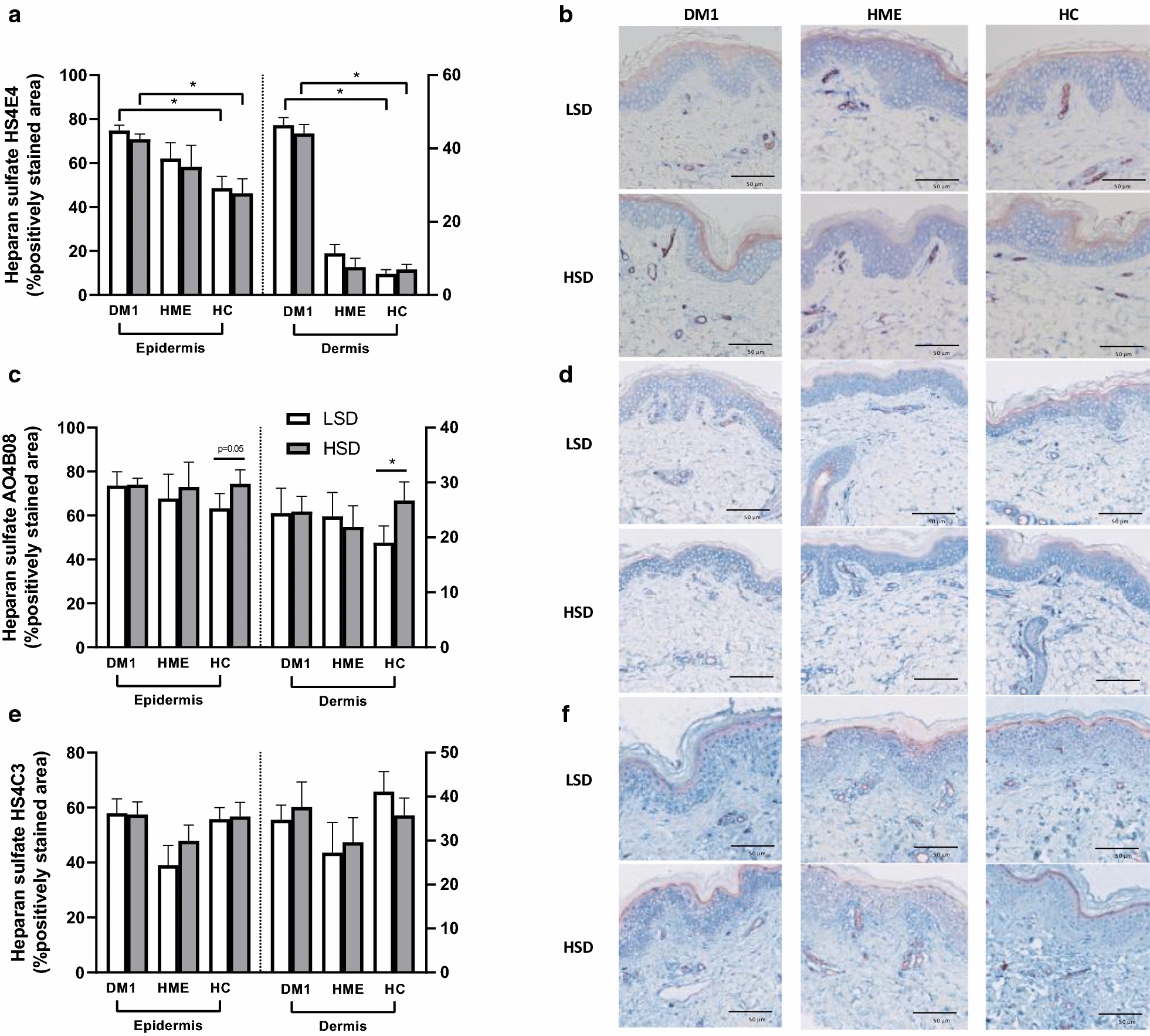
Expression of heparan sulfate in epidermal and dermal skin. **a**, **c**, **e** Semi- quantitative analysis. **b** Generally, DM1 showed a higher expression of low sulfated heparan sulfate (blue) than HC. However, the staining extent of low sulfated heparan sulfate (blue) in DM1 was diffuse and of low intensitiy. **d** HSD increased the expression of moderately sulfated heparan sulfate (AO4B08, blue) in the dermis of HC. **f** For high sulfated heparan sulfate (HS4C3, blue) no differences could be observed between groups or between diets. We observed that high sulfated heparan sulfate showed a nuclear staining pattern. Blue represents HS4E4, AO4B08, or HS4C3, respectively. Purple represents Ulex Europaeus Agglutinin I (UEA I). LSD, low sodium diet. HSD, high sodium diet. DM1, type 1 diabetes. HME, hereditary multiple exostosis. HC, healthy controls. *n* = 8 (DM1), *n* = 7 (HME), *n* = 12 (HC). *P < 0.05. Data are presented as individual data with mean (SEM). Data were tested with a paired t-test or Wilcoxon test, as appropriate, to compare two diets, and with an unpaired t-test or Kolmogorov–Smirnov test, as appropriate, to compare two patient groups

For dermal heparan sulfate, the presence of IdoA2S-GlcNS6S as detected by AO4B08, used as marker for moderately sulfated heparan sulfate, increased after HSD in the dermis of healthy controls (Fig. 6). We observed a strong increase of AO4B08 staining in both the extent of cellular staining in papillary dermis and the endothelium upon HSD (Fig. 6d) (Additional file 1: Figure S5). In DM1 and HME, the presence of the AO4B08 heparan sulfate domain showed no specific salt-induced alterations in the dermis. The extent of staining for HS4E4 (used as marker for relatively low sulfated heparan sulfate domains) and HS4C3 (IdoA2S-GlcNS3S6S, used as marker for highly sulfated heparan sulfate domains) in the dermis showed no clear sodium-induced alterations in either of the groups. We semi-quantitatively measured a higher percentage of positively stained area of HS4E4 in the dermis of DM1, however this could not be confirmed qualitatively (Additional file 1: Figure S6).

**Fig. 7:**
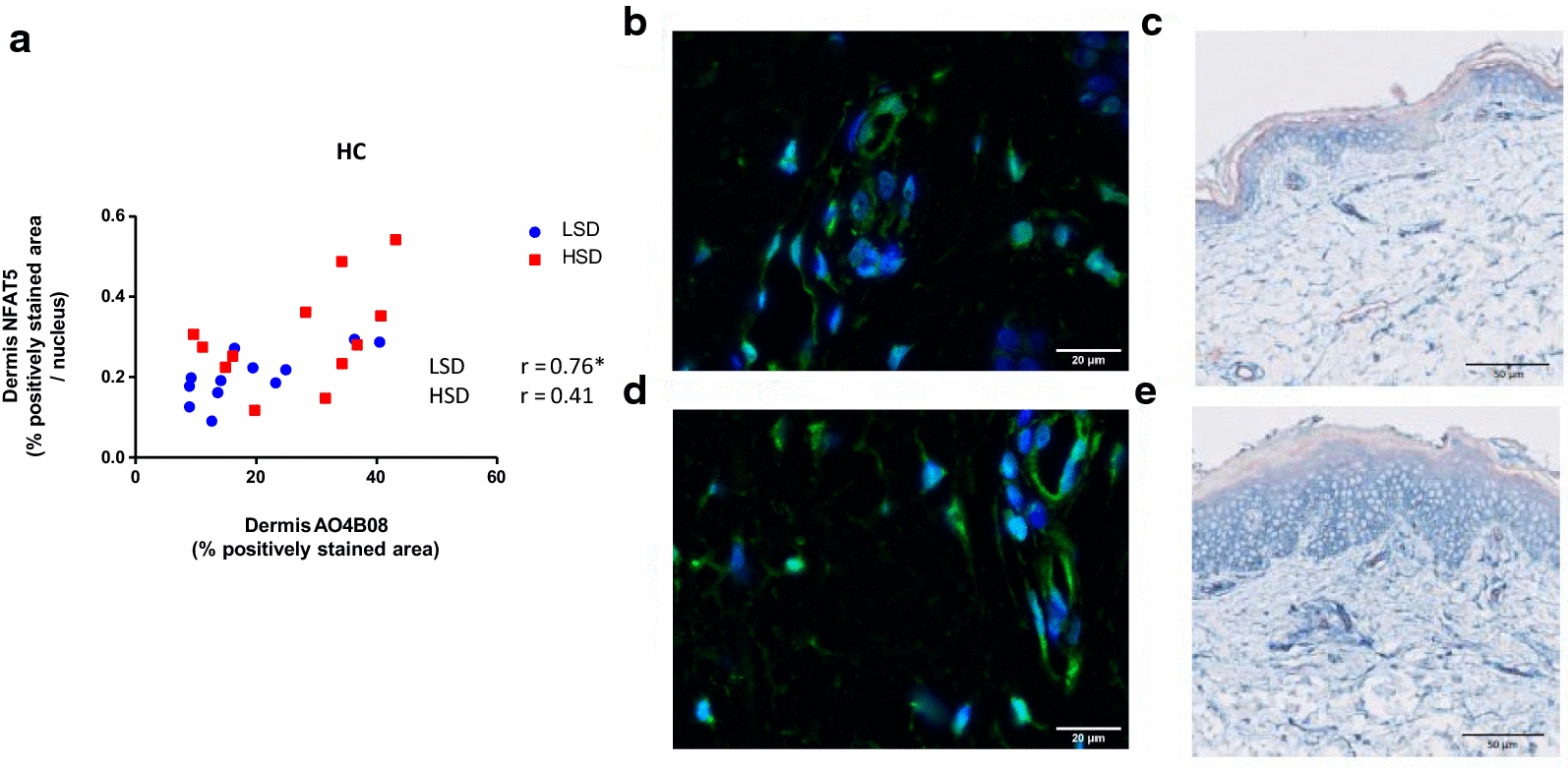
Correlation between NFAT5 and moderately sulfated heparan sulfate (AO4B08). **a** Linear regression graph showing the correlation between NFAT5 and AO4B08. NFAT5 expression in the dermis of healthy controls showed a significant positive correlation during LSD (r = 0.76, p = 0.004) with AO4B08. **b**, **c** Low expression of dermis NFAT5 in cytoplasm of endothelium (green staining in B) was accompanied by low expression of moderately sulfated heparan sulfate (AO4B08) (blue staining in C) in the dermis. **d**, **e** High expression of dermis NFAT5 in both endothelium and in nuclei and cytoplasm of perivascular leukocytes (green staining in D) was accompanied by a high content of moderately sulfated heparan sulfate in the dermis (blue staining in E). * marks a significant correlation. HC, healthy controls. LSD, low sodium diet. HSD, high sodium diet. NFAT5, nuclear factor of activated T-cells 5. *n* = 12. Data are presented as individual data. Data were tested with Spearman’s correlation coefficient

With our semi-quantitative analysis, we observed HSD-induced changes in the dermis of healthy controls, where both NFAT5 and the AO4B08 domain of heparan sulfate (moderately sulfated heparan sulfate domains) content increased per surface area. For both, this mainly resulted from an increased staining pattern in endothelial cells. Semi-quantitative results of NFAT5 and moderately sulfated heparan sulfate domains showed a positive correlation during LSD (n = 12, r = 0.76, p = 0.004) (Fig. 7).

**Fig. 8:**
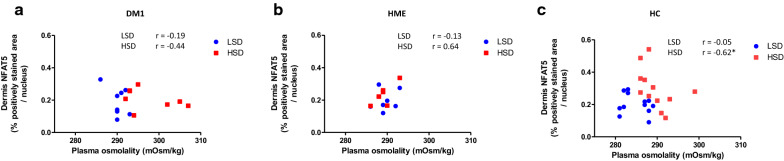
Negative correlation between NFAT5 expression in dermis and plasma osmolality in HC during HSD. **a**, **b** In DM1 and HME patients no correlation between dermis NFAT5 and plasma osmolality could be observed in HSD nor LSD. **c** NFAT5 expression in the dermis of healthy controls showed a significant negative correlation (r = −0.617, p = 0.032) with plasma osmolality during HSD. * marks a significant correlation. LSD, low sodium diet. HSD, high sodium diet. DM1, type 1 diabetes. HME, hereditary multiple exostosis. HC, healthy controls. NFAT5, nuclear factor of activated T-cells 5. Data are presented as individual data. Data were tested with Spearman’s correlation coefficient

To assess the association between the observed skin cellular response to HSD and body osmoregulation in healthy controls, we tested correlations between dermal NFAT5 expression and plasma and urine variables affected by HSD (Table 2). This showed that in healthy controls, NFAT5 expression was negatively correlated with plasma osmolality during HSD, while in DM1 and HME patients this correlation was absent (Fig. 8).

## Discussion

We investigated whether patients with either acquired or genetically determined GAG disturbances exhibit altered sodium and water homeostasis. We demonstrated that in response to hypertonicity, healthy volunteers showed an increase in both NFAT5 expression as well as in the abundance of the AO4B08 epitope of heparan sulfate (used as marker for moderately sulfated heparan sulfate domains), while these changes were absent in DM1 and HME subjects. Whilst in healthy volunteers skin GAG changes were present in the dermis, an increase in epidermal dermatan sulfate was observed in HME subjects. The absence of cellular osmoregulatory responses to chronic salt loading in DM1 subjects might reflect a reduced capacity for tissue sodium loading which can explain the unexpectedly high increase in plasma sodium concentration following hypertonic saline infusion.

The finding that HSD increases NFAT5 expression and influences the sulfation degree of heparan sulfates in the dermis of healthy controls extends previous observations in healthy volunteers, in which a 1-week HSD increases skin sodium, macrophages, vascular endothelial growth factor-C (VEGF-C), and lymphatics [18, 39]. The increase of the osmotically sensitive transcription factor NFAT5 reflects electrolyte accumulation in excess of water, resulting in local hypertonicity as shown by animal studies of the group of Titze et al. [10, 14, 15, 17, 40] Moreover, in other studies of this group, HSD led to increases in skin sodium, macrophage influx, and lymphatic expansion via expression of VEGF-C in macrophages [10, 14, 15, 17, 40]. These changes coincided with increases in GAG content and increases in specific GAG chain elongation enzymes, which are crucial for dermatan sulfate and chondroitin sulfate synthesis [14]. A cross-sectional study with human abdominal tissue obtained during surgery showed close correlations with sodium content and GAG content [16]. While these data, together with in vitro experiments showing GAGs are able to bind sodium, indicate that GAGs may have a key role in tissue sodium buffering [12, 13], data of skin GAGs responses to sodium loading in humans were lacking. Furthermore, determination of which types of GAGs this would apply to still remains [16]. We now demonstrated that HSD specifically increases the AO4B08 domain of sulfated heparan sulfate, interpreted as moderately sulfated heparan sulfate, in healthy volunteers, and that expression of NFAT5 significantly correlates with the amount of moderately sulfated heparan sulfate.

We observed HSD-induced NFAT5 and GAG changes in the dermis of healthy volunteers, whereas epidermal changes in this group were not as clearly apparent. We observed increased extent of nuclear NFAT5 in endothelial cells, implicating increased activation of NFAT5 target genes. In the dermis, endothelial cells are the first cells undergoing increased osmotic stress during increased intravascular sodium concentrations. In this context, it is remarkable that DM1 and HME did not show cellular responses to osmotic stress during HSD. In a previous study showing increased skin sodium content after HSD, the exact localization of skin sodium accumulation failed to be determined [18]. Also, specialized ^23^Na-MRI, which visualizes sodium accumulation in the skin, was not able to distinguish sodium accumulation in the epidermis from dermal sodium accumulation [41]. Our observations indicate a role for the dermis in skin sodium buffering during HSD. However, detection of accumulation of skin sodium ions at high spatial resolution is required to substantiate these findings furthermore.

In DM1 patients, no HSD-associated skin changes in NFAT5 or GAGs could be observed. This is in line with the absence of HSD-induced skin macrophage and lymphatic changes in these patients (in contrast to healthy subjects, who showed macrophage increases and lymphatic expansion in response to HSD) [39]. We previously hypothesized that this possibly reflects the absence of sodium influx, for example as a consequence of an already saturated buffer due to high skin sodium content at baseline. In the present study, higher urine (low sulfated) heparan sulfate excretion were found at baseline in DM1 compared to controls. Furthermore, DM1 patients showed no HSD-induced fluctuations in highly sulfated skin GAG expression (Alcian Blue staining), while this was observed in healthy controls. Although this may fit with higher sodium content in this patient group, as has been demonstrated earlier in type 2 diabetes patients, this is yet to be established for type 1 diabetes [42, 43]. Also, our acute sodium loading experiment suggests reduced sodium storage capacity in DM1 patients, as plasma sodium increase was higher in this patient group compared to healthy controls and higher than expected according to the Edelman-based formulas. Edelman et al. empirically showed that plasma water sodium = 1.11 * (total exchangeable body sodium + potassium)/total body water—25.6 [44]. Since our experimental set-up ensured that changes induced by hypertonic saline infusion in both total exchangeable body sodium + potassium cations and total body water were similar in the three groups, this would either mean altered slope (1.11) or altered y-intercept (−25.6) in DM1 patients when adopting the classical two-compartment view of sodium and water homeostasis. The two factors that determine the slope of the Edelman experiments—i.e., the Gibbs-Donnan equilibrium and osmotic coefficient of sodium—are not different in DM1 patients, nor are the first two of the three factors that determine the y-intercept—i.e., respectively, plasma potassium, osmotically active non-sodium and non-potassium osmoles (such as glucose and urea), and non-osmotically stored sodium and potassium [45]. Therefore, both increased plasma sodium and the lack of HSD-associated skin changes in DM1 patients may reflect reduced skin sodium storage capacity. It is to be noted that we previously showed that there were no considerable differences in plasma renin and aldosterone responses between DM1 patients and controls, thereby ruling these factors out as potential underlying causes for the other observed differences [46].

Considering the increase of epidermis dermatan sulfate in response to chronic sodium load, one might speculate that HME patients seem to compensate in this way for their disturbance in heparan sulfate. Additionally, urinary dermatan sulfate in HME patients is higher than in controls. This might represent a compensatory mechanism, consistent with the idea that reduction of one type of GAG could affect the production of the other, like the finding that somatic cells bearing mutations in EXT1 show increased chondroitin sulfate synthesis [47] and the observation that EXT1^±^EXT2^±^ mice (mouse model for HME) show increased dermatan sulfate content compared to wildtypes [25]. Furthermore, in the urine of DM1 patients and HME patients the dermatan sulfate disaccharide D0a10 as present. D0a10 is a disaccharide that is ordinarily absent in healthy individuals, but is known to be found in mucopolysaccharidosis patients, who are characterized by deficiency or malfunctioning of lysosomal enzymes for GAG breakdown [48]. It remains unclear whether this structural alteration has effect on sodium binding capacity. In DM1, increased expression of heparanase and possibly sulfatases may be involved in modulation of HS, as has been documented for diabetic nephropathy [49, 50]. The present acute sodium loading experiment indicates that acute sodium buffering capacity in HME patients might not be different compared to controls, despite their disturbance in heparan sulfate polymerization, potentially explained by compensatory dermatan sulfate. As experimental studies showed that skin sodium accumulation was paralleled by elevated GAG content, one might expect that HSD-induced increase in skin dermatan sulfate coincides with increased skin sodium content [15]. However, we were not able to observe a significant increase in dermis dermatan sulfate and NFAT5 (as a reflection of increased skin sodium/osmolarity) in this patient group, possibly due to a power problem.

We are—to our knowledge—the first to translate existing preclinical hypotheses on the link between sodium and GAGs by studying osmoregulation responses after sodium loading in patients with GAG alterations. The indications for a link between GAG disturbances and altered osmoregulation responses motivate further research to confirm causality, as this will have significant clinical impact on the pathophysiology and management of dysnatremias and its associated adverse outcomes in clinical practice [2]. However, certain limitations deserve consideration. First, skin sodium content could not be directly assessed in this study, due to the limited amount of skin material that we could obtain from participants. We did, however, use NFAT5 expression as reflection of osmolarity, because of its known responsiveness to osmolarity increases (hence its alternative name ‘tonicity-responsive enhancer binding protein’) and known associations of NFAT5 loci with plasma osmolality on population level [51]. Also, interpretation of GAG measurements can be complex. Although we are the first to provide a closer look into specific types and specific sulfation grades of skin GAGs in the context of salt loading, it is impossible to perform stainings for every existing sulfation grade. Therefore, the presently studied sulfation grades of GAGs are not all-encompassing. Finally, although this study brings translation of the link between GAGs and sodium one step closer, the co-occurrence of GAG alterations and indications for altered sodium homeostasis does not prove a causal relationship. For this, we still rely on studies that assess sodium homeostasis after intervening with GAG status. Sulodexide is an example of a GAG intervention since its main component is synthetic heparan sulfate. This drug was previously shown to restore the endothelial surface layer [52] and to lower blood pressure with borderline significant effects on plasma sodium [53]. Although these prospective data may point towards increased sodium buffering capacity, definitive proof is still lacking and assessment of sodium homeostasis/sodium buffering capacity after GAG interventions is warranted.

## Conclusions

Two patient groups with GAG alterations—DM1 and HME patients—show altered osmoregulation responses to a sodium load, with indications for reduced sodium capacity in DM1 patients. Future research with interventions targeting GAGs is necessary to establish whether this relation is causal, which will significantly impact osmoregulation disorders and their management.

## Supplementary Information

**Additional file 1: Table S1**. Absolute changes in other laboratory parameters during the acute sodium experiment in DM1 patients, HME patients and healthy controls. **Figure S1.** Absolute changes of other laboratory parameters compared to baseline in DM1 patients, HME patients and healthy controls. **Figure S2.** Sodium homeostasis after acute hypertonic saline infusion. A-C) Observed and expected sodium/cation levels in plasma and urine in DM1 patients (A), HME patients (B), and healthy controls (C). **Figure S3.** Alcian Blue pH1 staining of the skin. **Figure S4**. Alcian Blue pH2.7 staining of the skin. **Figure S5.** Staining of the AO4B08 domain of heparan sulfate in the skin. **Figure S6.** Staining of the HS4E4 domain of heparan sulfate in the skin.** Figure S7.** Correlation between epidermal NFAT5 and epidermal GD3A12 expression in HME patients. **Appendix 1.** Adrogue-Madias formula and Nguyen-Kurtz formula. **Appendix 2**. Detailed description of performed histochemistry and immunostaining

## Data Availability

All data generated and/or analysed during the current study are available from the corresponding author on reasonable request.

## Abbreviations

BP: Blood pressure
DM1: Type 1 diabetes
GAGs: Glycosaminoglycans
HC: Healthy controls
HME: Hereditary multiple exostosis
HSD: High sodium diet
LSD: Low sodium diet
NFAT5: Nuclear factor of activated t-cells 5
TonEBP: Tonicity-responsive enhancer binding protein
VEGF-C: Vascular endothelial growth factor-C

## References

[1] Sterns RH (2015). Disorders of plasma sodium–causes, consequences, and correction. N Engl J Med.

[2] Darmon M, Diconne E, Souweine B, Ruckly S, Adrie C, Azoulay E, Clec'h C, Garrouste-Orgeas M, Schwebel C, Goldgran-Toledano D (2013). Prognostic consequences of borderline dysnatremia: pay attention to minimal serum sodium change. Crit Care.

[3] Frenkel WN, van den Born BJ, van Munster BC, Korevaar JC, Levi M, de Rooij SE (2010). The association between serum sodium levels at time of admission and mortality and morbidity in acutely admitted elderly patients: a prospective cohort study. J Am Geriatr Soc.

[4] Sun L, Hou Y, Xiao Q, Du Y (2017). Association of serum sodium and risk of all-cause mortality in patients with chronic kidney disease: a meta-analysis and sysematic review. Sci Rep.

[5] Waite MD, Fuhrman SA, Badawi O, Zuckerman IH, Franey CS (2013). Intensive care unit-acquired hypernatremia is an independent predictor of increased mortality and length of stay. J Crit Care.

[6] Hu J, Wang Y, Geng X, Chen R, Zhang P, Lin J, Teng J, Zhang X, Ding X (2017). Dysnatremia is an independent indicator of mortality in hospitalized patients. Med Sci Monit.

[7] Hoorn EJ, Zietse R (2008). Hyponatremia revisited: translating physiology to practice. Nephron Physiol.

[8] Engberink ORH, Rorije NM, van der Heide HJJ, van den Born BJ, Vogt L (2015). Role of the vascular wall in sodium homeostasis and salt sensitivity. J Am Soc Nephrol.

[9] Wenstedt EFE, Olde Engberink RHG, Vogt L (2018). Sodium handling by the blood vessel wall: critical for hypertension development. Hypertension.

[10] Titze J, Lang R, Ilies C, Schwind KH, Kirsch KA, Dietsch P, Luft FC, Hilgers KF (2003). Osmotically inactive skin Na+ storage in rats. Am J Physiol Renal Physiol.

[11] Olde Engberink RH, Rorije NM, van den Born BH, Vogt L (2017). Quantification of nonosmotic sodium storage capacity following acute hypertonic saline infusion in healthy individuals. Kidney Int.

[12] Farber SJ, Schubert M, Schuster N (1957). The binding of cations by chondroitin sulfate. J Clin Invest.

[13] Siegel G, Walter A, Kauschmann A, Malmsten M, Buddecke E (1996). Anionic biopolymers as blood flow sensors. Biosens Bioelectron.

[14] Titze J, Shakibaei M, Schafflhuber M, Schulze-Tanzil G, Porst M, Schwind KH, Dietsch P, Hilgers KF (2004). Glycosaminoglycan polymerization may enable osmotically inactive Na+ storage in the skin. Am J Physiol Heart Circ Physiol.

[15] Machnik A, Neuhofer W, Jantsch J, Dahlmann A, Tammela T, Machura K, Park JK, Beck FX, Muller DN, Derer W (2009). Macrophages regulate salt-dependent volume and blood pressure by a vascular endothelial growth factor-C-dependent buffering mechanism. Nat Med.

[16] Fischereder M, Michalke B, Schmoeckel E, Habicht A, Kunisch R, Pavelic I, Szabados B, Schonermarck U, Nelson P, Stangl M: Sodium storage in human tissues is mediated by glycosaminoglycan expression. Am J Physiol Renal Physiol 2017: ajprenal 00703 02016. 10.1152/ajprenal.00703.2016 28446462

[17] Titze J, Krause H, Hecht H, Dietsch P, Rittweger J, Lang R, Kirsch KA, Hilgers KF (2002). Reduced osmotically inactive Na storage capacity and hypertension in the Dahl model. Am J Physiol Renal Physiol.

[18] Selvarajah V, Maki-Petaja KM, Pedro L, Bruggraber SFA, Burling K, Goodhart AK, Brown MJ, McEniery CM, Wilkinson IB (2017). Novel mechanism for buffering dietary salt in humans: effects of salt loading on skin sodium, vascular endothelial growth factor c, and blood pressure. Hypertension.

[19] Hiebert LM (2017). Proteoglycans and diabetes. Curr Pharm Des.

[20] Gowd V, Gurukar A, Chilkunda ND (2016). Glycosaminoglycan remodeling during diabetes and the role of dietary factors in their modulation. World J Diabetes.

[21] Cechowska-Pasko M, Palka J, Bankowski E (1996). Decrease in the glycosaminoglycan content in the skin of diabetic rats. The role of IGF-I, IGF-binding proteins and proteolytic activity. Mol Cell Biochem.

[22] De Muro P, Fresu P, Formato M, Tonolo G, Mameli M, Maioli M, Sanna GM, Cherchi GM (2002). Urinary glycosaminoglycan and proteoglycan excretion in normoalbuminuric patients with type 1 diabetes mellitus. J Nephrol.

[23] Zak BM, Crawford BE, Esko JD (2002). Hereditary multiple exostoses and heparan sulfate polymerization. Biochim Biophys Acta.

[24] Mooij HL, Cabrales P, Bernelot Moens SJ, Xu D, Udayappan SD, Tsai AG, van der Sande MA, de Groot E, Intaglietta M, Kastelein JJ (2014). Loss of function in heparan sulfate elongation genes EXT1 and EXT 2 results in improved nitric oxide bioavailability and endothelial function. J Am Heart Assoc.

[25] Olde Engberink RHG, de Vos J, van Weert A, Zhang Y, van Vlies N, van den Born BH, Titze JM, van Bavel E, Vogt L (2019). Abnormal sodium and water homeostasis in mice with defective heparan sulfate polymerization. PLoS ONE.

[26] Rorije NMG, Olde Engberink RHG, Chahid Y, van Vlies N, van Straalen JP, van den Born BH, Verberne HJ, Vogt L: Microvascular Permeability after an Acute and Chronic Salt Load in Healthy Subjects: A Randomized Open-label Crossover Intervention Study. Anesthesiology. 2017:352–360. 10.1097/ALN.0000000000001989 29206647

[27] Wouda RD, Dekker SEI, Reijm J, Engberink ORHG, Vogt L (2019). Effects of water loading on observed and predicted plasma sodium, and fluid and urine cation excretion in healthy individuals. Am J Kidney Dis.

[28] Nguyen MK, Kurtz I (2003). A new quantitative approach to the treatment of the dysnatremias. Clin Exp Nephrol.

[29] Adrogue HJ, Madias NE (1997). Aiding fluid prescription for the dysnatremias. Intensive Care Med.

[30] Hillier TA, Abbott RD, Barrett EJ (1999). Hyponatremia: evaluating the correction factor for hyperglycemia. Am J Med.

[31] Langereis EJ, van Vlies N, Church HJ, Geskus RB, Hollak CE, Jones SA, Kulik W, van Lenthe H, Mercer J, Schreider L (2015). Biomarker responses correlate with antibody status in mucopolysaccharidosis type I patients on long-term enzyme replacement therapy. Mol Genet Metab.

[32] Lawrence R, Lu H, Rosenberg RD, Esko JD, Zhang L (2008). Disaccharide structure code for the easy representation of constituent oligosaccharides from glycosaminoglycans. Nat Methods.

[33] Lensen JF, Wijnhoven TJ, Kuik LH, Versteeg EM, Hafmans T, Rops AL, Pavao MS, van der Vlag J, van den Heuvel LP, Berden JH, van Kuppevelt TH (2006). Selection and characterization of a unique phage display-derived antibody against dermatan sulfate. Matrix Biol.

[34] Ten Dam GB, Yamada S, Kobayashi F, Purushothaman A, van de Westerlo EM, Bulten J, Malmstrom A, Sugahara K, Massuger LF, van Kuppevelt TH (2009). Dermatan sulfate domains defined by the novel antibody GD3A12, in normal tissues and ovarian adenocarcinomas. Histochem Cell Biol.

[35] Ten Dam GB, Kurup S, van de Westerlo EM, Versteeg EM, Lindahl U, Spillmann D, van Kuppevelt TH (2006). 3-O-sulfated oligosaccharide structures are recognized by anti-heparan sulfate antibody HS4C3. J Biol Chem.

[36] Kurup S, Wijnhoven TJ, Jenniskens GJ, Kimata K, Habuchi H, Li JP, Lindahl U, van Kuppevelt TH, Spillmann D (2007). Characterization of anti-heparan sulfate phage display antibodies AO4B08 and HS4E4. J Biol Chem.

[37] Schneider CA, Rasband WS, Eliceiri KW (2012). NIH image to ImageJ: 25 years of image analysis. Nat Methods.

[38] Ruifrok AC, Johnston DA (2001). Quantification of histochemical staining by color deconvolution. Anal Quant Cytol Histol.

[39] Wenstedt EFE, Engberink R, Rorije NMG, van den Born BH, Claessen N, Aten J, Vogt L (2019). Salt-sensitive blood pressure rise in type 1 diabetes patients is accompanied by disturbed skin macrophage influx and lymphatic dilation-a proof-of-concept study. Transl Res.

[40] Schafflhuber M, Volpi N, Dahlmann A, Hilgers KF, Maccari F, Dietsch P, Wagner H, Luft FC, Eckardt KU, Titze J (2007). Mobilization of osmotically inactive Na(+) by growth and by dietary salt restriction in rats. Am J Physiol Renal Physiol.

[41] Machnik A, Dahlmann A, Kopp C, Goss J, Wagner H, van Rooijen N, Eckardt KU, Muller DN, Park JK, Luft FC (2010). Mononuclear phagocyte system depletion blocks interstitial tonicity-responsive enhancer binding protein/vascular endothelial growth factor C expression and induces salt-sensitive hypertension in rats. Hypertension.

[42] Kopp C, Linz P, Wachsmuth L, Dahlmann A, Horbach T, Schofl C, Renz W, Santoro D, Niendorf T, Muller DN (2012). (23)Na magnetic resonance imaging of tissue sodium. Hypertension.

[43] Kopp C, Linz P, Maier C, Wabel P, Hammon M, Nagel AM, Rosenhauer D, Horn S, Uder M, Luft FC (2018). Elevated tissue sodium deposition in patients with type 2 diabetes on hemodialysis detected by (23)Na magnetic resonance imaging. Kidney Int.

[44] Kannenkeril D, Karg MV, Bosch A, Ott C, Linz P, Nagel AM, Uder M, Schmieder RE (2019). Tissue sodium content in patients with type 2 diabetes mellitus. J Diabetes Complications.

[45] Edelman IS, Leibman J, O'Meara MP, Birkenfeld LW (1958). Interrelations between serum sodium concentration, serum osmolarity and total exchangeable sodium, total exchangeable potassium and total body water. J Clin Invest.

[46] Nguyen MK, Kurtz I (2004). Determinants of plasma water sodium concentration as reflected in the Edelman equation: role of osmotic and Gibbs-Donnan equilibrium. Am J Physiol Renal Physiol.

[47] Wenstedt EFE, Rorije NMG, Olde Engberink RHG, van der Molen KM, Chahid Y, Danser AHJ, van den Born BH, Vogt L (2020). Effect of high-salt diet on blood pressure and body fluid composition in patients with type 1 diabetes: randomized controlled intervention trial. BMJ Open Diabetes Res Care.

[48] Wei G, Bai X, Gabb MM, Bame KJ, Koshy TI, Spear PG, Esko JD (2000). Location of the glucuronosyltransferase domain in the heparan sulfate copolymerase EXT1 by analysis of Chinese hamster ovary cell mutants. J Biol Chem.

[49] de Ru MH, van der Tol L, van Vlies N, Bigger BW, Hollak CE, Ijlst L, Kulik W, van Lenthe H, Saif MA, Wagemans T (2013). Plasma and urinary levels of dermatan sulfate and heparan sulfate derived disaccharides after long-term enzyme replacement therapy (ERT) in MPS I: correlation with the timing of ERT and with total urinary excretion of glycosaminoglycans. J Inherit Metab Dis.

[50] van den Hoven MJ, Rops AL, Bakker MA, Aten J, Rutjes N, Roestenberg P, Goldschmeding R, Zcharia E, Vlodavsky I, van der Vlag J, Berden JH (2006). Increased expression of heparanase in overt diabetic nephropathy. Kidney Int.

[51] Garsen M, Rops AL, Rabelink TJ, Berden JH, van der Vlag J (2014). The role of heparanase and the endothelial glycocalyx in the development of proteinuria. Nephrol Dial Transplant.

[52] Boger CA, Gorski M, McMahon GM, Xu H, Chang YC, van der Most PJ, Navis G, Nolte IM, de Borst MH, Zhang W (2017). NFAT5 and SLC4A10 loci associate with plasma osmolality. J Am Soc Nephrol.

[53] Broekhuizen LN, Lemkes BA, Mooij HL, Meuwese MC, Verberne H, Holleman F, Schlingemann RO, Nieuwdorp M, Stroes ES, Vink H (2010). Effect of sulodexide on endothelial glycocalyx and vascular permeability in patients with type 2 diabetes mellitus. Diabetologia.

[54] Olde Engberink RH, Rorije NM, Lambers Heerspink HJ, De Zeeuw D, van den Born BJ, Vogt L (2015). The blood pressure lowering potential of sulodexide–a systematic review and meta-analysis. Br J Clin Pharmacol.

[55] Moher D, Hopewell S, Schulz KF, Montori V, Gotzsche PC, Devereaux PJ, Elbourne D, Egger M, Altman DG (2010). CONSORT 2010 explanation and elaboration: updated guidelines for reporting parallel group randomised trials. BMJ.

