# Supplementary material for: Distinct osmoregulatory responses to sodium loading in patients with altered glycosaminoglycan structure: a randomized cross-over trial
Source: J Transl Med. 2021 Jan 20;19:38. doi: 10.1186/s12967-021-02700-0 (PMC7816310; doi:10.1186/s12967-021-02700-0)
Supplement: Supplementary file 1 — Additional file 1: Table S1. Absolute changes in other laboratory parameters during the acute sodium experiment in DM1 patients, HME patients and healthy controls. Figure S1. Absolute changes of other laboratory parameters compared to baseline in DM1 patients, HME patients and healthy controls. Figure S2. Sodium homeostasis after acute hypertonic saline infusion. A-C) Observed and expected sodium/cation levels in plasma and urine in DM1 patients (A), HME patients (B), and healthy controls (C). Figure S3. Alcian Blue pH1 staining of the skin. Figure S4. Alcian Blue pH2.7 staining of the skin. Figure S5. Staining of the AO4B08 domain of heparan sulfate in the skin. Figure S6. Staining of the HS4E4 domain of heparan sulfate in the skin. Figure S7. Correlation between epidermal NFAT5 and epidermal GD3A12 expression in HME patients. Appendix 1. Adrogue-Madias formula and Nguyen-Kurtz formula. Appendix 2. Detailed description of performed histochemistry and immunostaining [file 12967_2021_2700_MOESM1_ESM.docx]

**DISTINCT OSMOREGULATORY RESPONSES TO SODIUM LOADING IN PATIENTS WITH ALTERED GLYCOSAMINOGLYCAN STRUCTURE – A RANDOMIZED CROSS-OVER TRIAL**

**Supplementary index**

**Supplemental table 1**. Absolute changes in other laboratory parameters during the acute sodium experiment in DM1 patients, HME patients and healthy controls

**Supplemental figure 1.** Absolute changes of other laboratory parameters compared to baseline in DM1 patients, HME patients and healthy controls

**Supplemental figure 2.** Sodium homeostasis after acute hypertonic saline infusion.
A-C) Observed and expected sodium/cation levels in plasma and urine in DM1 patients (A), HME patients (B), and healthy controls

**Supplemental figure 3.** Alcian Blue pH1 staining of the skin

**Supplemental figure 4**. Alcian Blue pH2.7 staining of the skin

**Supplemental figure 5.** Staining of the AO4B08 domain of heparan sulfate in the skin

**Supplemental figure 6.** Staining of the HS4E4 domain of heparan sulfate in the skin

**Supplemental figure 7.** Correlation between epidermal NFAT5 and epidermal GD3A12 expression in HME patients

**Supplementary appendix 1.** Adrogue-Madias formula and Nguyen-Kurtz formula

**Supplementary appendix 2**. Detailed description of performed histochemistry and immunostaining

**SUPPLEMENTARY TABLE**

**Supplemental table 1. Absolute changes in other laboratory parameters during the acute sodium experiment in DM1 patients, HME patients and healthy controls**

Data are depicted as mean (SEM).

|  | **Type 1 diabetes patients  (n=8)** | | | **HME patients**  **(n=7)** | | | **Healthy controls  (n=11)** | | |
| --- | --- | --- | --- | --- | --- | --- | --- | --- | --- |
|  | **BL** | **2h** | **4h** | **BL** | **2h** | **4h** | **BL** | **2h** | **4h** |
| Total body water (L) | 45.3 (1.9) | 45.5  (1.9) | 45.4 (1.9) | 46.3 (1.9) | 46.4 (1.9) | 46.3 (1.9) | 44.5  (1.3) | 44.6 (1.2) | 44.9 (1.2) |
| Urine osmolality (mOsm/kg) | 417.1 (58.8) | 455.5 (46.4) | 538.0 (58.4) | 229.1 (22.7) | 323.7 (24.5) | 629.3 (52.6) | 299.8 (17.1) | 365.3 (28.2) | 679.2 (30.4) |
| Urine sodium (mmol/L) | 12.8 (3.8) | 45.6 (13.6) | 44.6 (7.9) | 12.4 (2.1) | 52.9 (14.1) | 119.7 (22.7) | 8.7  (1.2) | 42.3 (7.3) | 109.9 (7.7) |
| Urine potassium (mmol/L) | 54.6 (5.2) | 62.0  (8.1) | 79.9 (12.5) | 30.3 (5.4) | 38.7 (8.8) | 70.7 (8.9) | 40.1  (4.0) | 47.8 (5.4) | 68.6 (6.6) |
| Fractional sodium excretion (%) | 0.1 (0.03) | 0.3  (0.1) | 0.3 (0.1) | 0.2 (0.03) | 0.5  (0.2) | 0.5 (0.1) | 0.1  (0.01) | 0.4 (0.09) | 0.5 (0.08) |
| Free water clearance (mL/min) | -0.4 (0.2) | -1.1  (0.3) | -0.6 (0.1) | 0.5  (0.2) | -0.2  (0.2) | -0.6 (0.1) | -0.05 (0.1) | -0.4 (0.1) | -0.9 (0.1) |

**SUPPLEMENTARY FIGURES**

**Supplemental Figure 1. Absolute changes of other laboratory parameters compared to baseline in DM1 patients, HME patients and healthy controls.** Data are presented as mean (SEM).

**Supplemental Figure 2. Sodium homeostasis after acute hypertonic saline infusion.
A-C) Observed and expected sodium/cation levels in plasma and urine in DM1 patients (A), HME patients (B), and healthy controls (C).** Using the Adrogue-Madias and Nguyen-Kurtz formula, we calculated how much cations should be in the urine to account for the plasma sodium decreases that take place from 5 minutes after infusion and onwards. For clarity, only the Adrogue-Madias formula is depicted in the graphs. DM1, type 1 diabetes. HME, hereditary multiple exostosis. HC, healthy controls


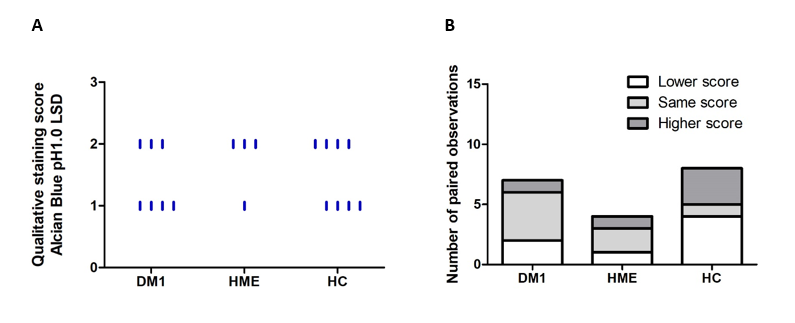


**Supplemental Figure 3. Alcian Blue pH1 staining of the skin. (A)** The overall extent of Alcian Blue staining at pH1 in the papillary matrix after LSD was similar in all groups. **(B)** Paired observations showed fluctuating Alcian Blue pH1 expression between both diets in the papillary matrix of HME and HC, however DM1 showed a constant staining pattern in the majority of the subjects. LSD, low sodium diet. DM1, type 1 diabetes. HME, hereditary multiple exostosis. HC, healthy controls.


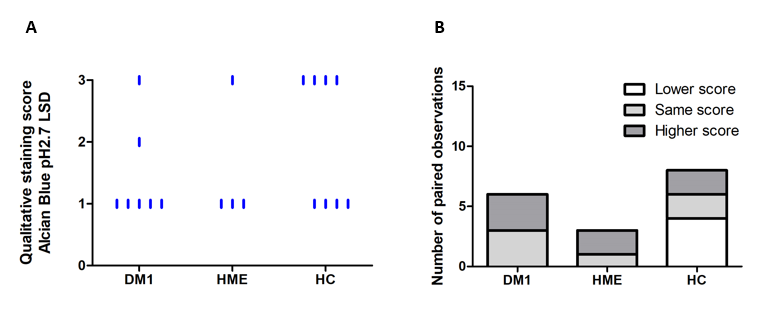


**Supplemental Figure 4. Alcian Blue pH2.7 staining of the skin. (A)** The overall extent of Alcian Blue staining at pH2.7 in the papillary matrix after LSD was similar in all groups. **(B)** Paired observations showed stable or increase or similar extent of Alcian Blue staining at pH2.7 in DM1, whereas in healthy controls a decrease was observed in the majority of the observations. LSD, low sodium diet. DM1, type 1 diabetes. HME, hereditary multiple exostosis. HC, healthy controls.


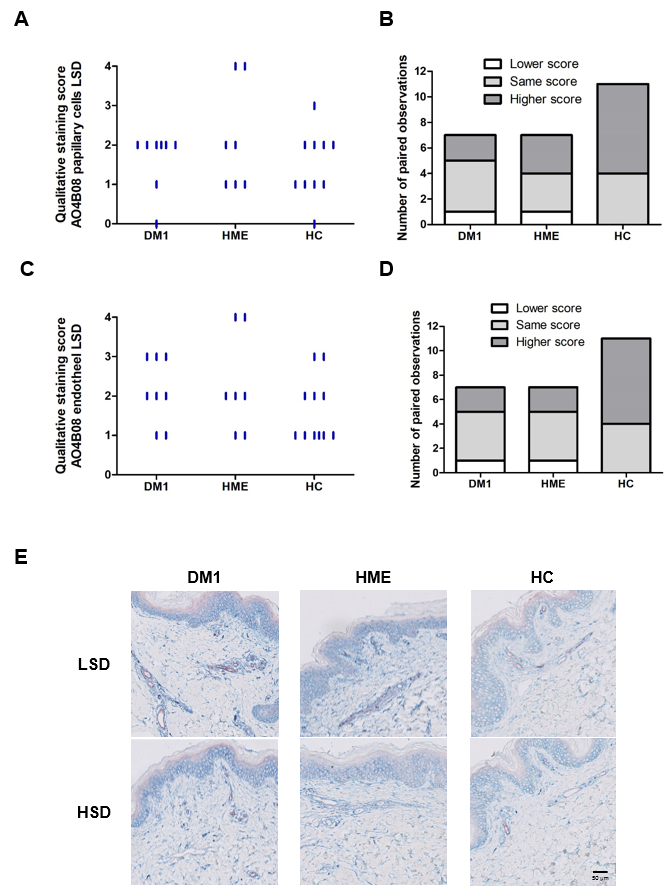


**Supplemental Figure 5. Staining of the AO4B08 domain of heparan sulfate in the skin. (A,C)** After LSD we were not able to observe clear differences in the extent of AO4B08 in either the cellular cells of the papillary dermis or the endothelium between the groups. **(B, D)**. Paired-observations showed an increase in both the extent of cellular staining in the papillary dermis and the endothelium upon HSD in healthy controls. In DM1 and HME no specific salt-induced alterations could be observed. **(E)** Paired histological images of AO4B08 (blue) in the dermis and epidermis showed an increased staining extent in the endothelium and papillary in healthy controls. Blue represents AO4B08. Purple represents Ulex Europaeus Agglutinin I (UEA I). LSD, low sodium diet. HSD, high sodium diet. DM1, type 1 diabetes. HME, hereditary multiple exostosis. HC, healthy controls.


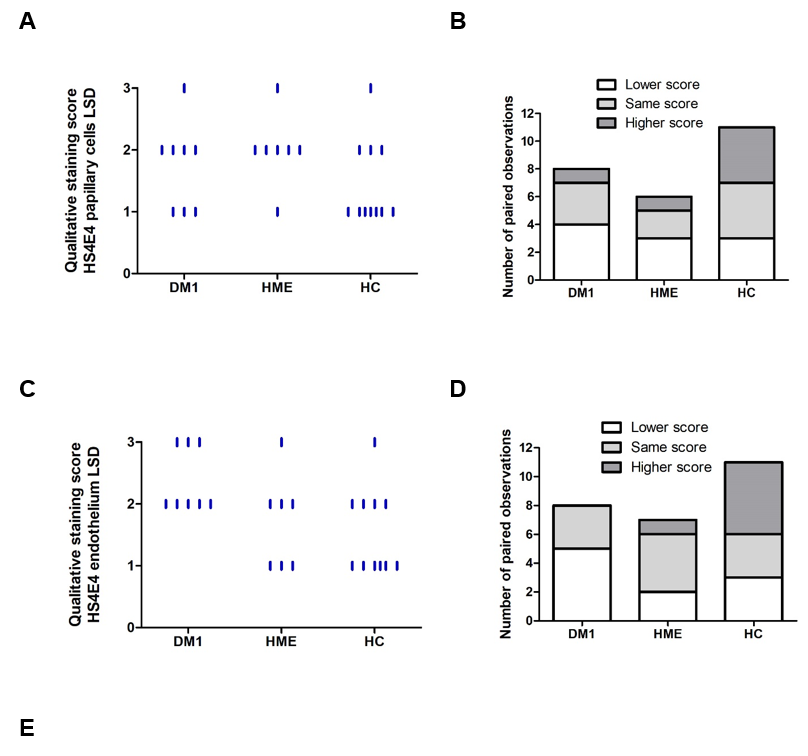


**Supplemental Figure 6. Staining of the HS4E4 domain of heparan sulfate in the skin. (A,C)** The quantitative staining score of HS4E4 after LSD for papillary cells and the endothelium shows a higher extent of HS4E4 expression in the endothelium of DM1. **(B, D)**. Paired-observations showed salt-induced fluctuations in all groups with no specific trend. **(E)** Paired histological images of HS4E4 (blue) in the dermis and epidermis showed an increased staining extent in the endothelium of DM1 upon LSD. In the epidermis no salt-induced changes could be observed. Blue represents HS4E4. Purple represents Ulex Europaeus Agglutinin I (UEA I). LSD, low sodium diet. HSD, high sodium diet. DM1, type 1 diabetes. HME, hereditary multiple exostosis. HC, healthy controls.

**Supplemental figure 7. Correlation between epidermal NFAT5 and epidermal GD3A12 expression in HME patients.** Linear regression graph showing the correlation between epidermal NFAT5 and epidermal GD3A12 expression. No correlation during HSD (r= -0.49, p=0.33) nor during LSD (r=0.25, p=0.59) could be observed. LSD, low sodium diet. HSD, high sodium diet. HME, hereditary multiple exostosis. NFAT5, nuclear factor of activated T-cells. Data were tested with Spearman’s correlation coefficient.

**SUPPLEMENTARY APPENDIX 1**

**Adrogue-Madias formula**

Original^1^:
∆ plasma Na^+^ (per liter_infusate_) = (Na^+^K^+^_infusate_ – plasma Na^+^_pre_) / (TBW + 1)

Fluid loss^2^:
∆ plasma Na^+^ = (plasma Na^+^_pre_ – Na^+^K^+^_fluid loss_) / (TBW – 1)

Rearranged fluid loss:
Na^+^K^+^_fluid_ loss = plasma Na^+^_pre_ – (∆ plasma Na^+^ * (TBW – 1))

**Nguyen-Kurtz formula**

Original^3^:
plasma Na^+^_post_ = ((plasma Na^+^_pre_ + 23.8) * TBW) + (1.03 * (Na^+^K^+^_input_) – (Na^+^K^+^_output_)) /
(TBW + ∆volume) – 23.8

Rearranged:
(Na^+^K^+^_input_) – (Na^+^K^+^_output_) = ((plasma Na^+^_pre_ + 23.8) * TBW) – ((23.8 + plasma Na^+^_post_) * (TBW + ∆volume)) / –1.03

**References**

1. Adrogue HJ, Madias NE. Aiding fluid prescription for the dysnatremias. Intensive Care Med 1997;23:309-16.

2. Adrogue HJ, Madias NE. The challenge of hyponatremia. J Am Soc Nephrol 2012;23:1140-8. doi: 10.681/ASN.2012020128. Epub 2012 May 24.

3. Nguyen MK, Kurtz I. A new quantitative approach to the treatment of the dysnatremias. Clin Exp Nephrol 2003;7:125-37.

**SUPPLEMENTARY APPENDIX 2**

**Immunohistochemistry**

Skin biopsies obtained from the upper medial forearm were obtained and subsequently embedded in paraffin. Sections of 4 micrometers thick were cut, and deparaffinized in xylene and rehydrated in graded alcohol series.

*NFAT5*

For immunohistochemical staining of NFAT5, the tissue samples were rinsed 5 times in demi water after rehydration. Next, they were cooked for 20 minutes at 100℃ in an autoclave (Prestige medical Classic, Northridge, CA, USA) in a Tris-EDTA (pH9) solution containing 10 mM UltraPure Tris (Invitrogen, Carlsbad, CA, USA) and 1 mM Titriplex III (Merck, Keniworth, NJ, USA). After cooling down to room temperature, tissue samples were once rinsed in PBS, followed by rinsing in PBS Tween20 0.01%. Then, tissue samples were blocked with 10ml of a solution containing PBS Tween20 0.01% and 1% bovine serum albumin (BSA). All tissue samples were incubated with a mixture of Rabbit IgG anti-NFAT5 (ThermoFisher; dilution 1:500) overnight at 4°C. Afterwards the sections were three times three minutes washed in PBS Tween20 0.01% and incubated for 30 minutes with a mixture of AlexaFluor488-conjugated Goat anti-Rabbit IgG antibodies (Jackson ImmunoResearch, Cambridgeshire, UK, dilution 1:200) to visualize anti-NFAT5 antibodies and Hoechst 33342 nuclear stain (thermoFisher, Waltham, Ma, USA, dilution 1:20000) for visualization of the nuclei. For staining of NFAT5, all antibodies were diluted in a mixture of PSB Tween 0.01% and bovine serum albumin (BSA) 0.1%. Finally, the tissue samples were mounted using Aqua Poly/Mount (Polysciences, Inc., Warrington, PA, USA) and dried for 24 hours at room temperature.

*Glycosaminoglycans*

For staining of glycosaminoglycans, the tissue samples were washed 3 times in Phosphate Buffered Saline (PBS) for 3 minutes and a peroxidase activity blocker (0,1% NaN3, 0,3% H2O2) was added for 10 minutes. The biopsies were washed 3 times in water, before they were cooked in an autoclave (Prestige medical Classic, Northridge, CA, USA) at 100℃ for 10 minutes in a Tris-EDTA (pH9) solution containing 10 mM UltraPure Tris (Invitrogen, Carlsbad, CA, USA) and 0.1 mM Titriplex III (Merck, Keniworth, NJ, USA). The tissue samples were incubated with one of the primary VSV-tagged antibodies against dermatan sulfate (LKN1 and GD3A12) or heparan sulfate (HS4C3, AO4B08 and HS4E4) (Radboud University, Nijmegen, the Netherlands; dilution 1:10) overnight at 4℃. For dermatan sulfate, the LKN1 antibody recognizes the expression of the 4/2.4-di-O-sulfated dermatan sulfate domain. The GD3A12 antibody recognizes the expression of the IdoA-Gal-NAc4S dermatan sulfate domain. These phage displayed-derived antibodies against dermatan sulfate were selected as described by Lensen et al.^32^ The three heparan sulfate antibodies (HS4C3, AO4B08, HS4E4) each recognize specific sulfation motifs and ligand-binding sites in heparan sulfate chains. HS4C3 binds 3-O-sulfated heparan sulfate chains with preference for the fully sulfated IdoA2S0GlcNS3S6S domains^36^, AO4B08 binds N-sulfated, 2-O-sulfated, and 6-O-sulfated heparan sulfate chains, corresponding to the IdoA2S-GlcNS6S domain which lacks 3-O sulfatations^37^, and HS4E4 binds heparan sulfate domains containing both N-sulfation and N-acetylation and in general heparan sulfate chains with low sulfation grades since the presence of 6-O-sulfated sites inhibits binding of HS4E4.^37^ The bound antibodies were detected by Rabbit anti-VSV (Sigma-Aldrich, Saint Louis, MO, USA; dilution 1:1000), followed by incubation with BrightVision Poly-AP-conjugated goat IgG anti-Rabbit IgG (ImmunoLogic; dilution 1:1 with PBSTween 20 (0,01%); Sigma-Aldrich). The sections were stained for 25 minutes using PermaBlue Plus/AP (Diagnostics BioSystems, Pleasanton, CA, USA; dilution 1:50 with the product buffer) with two drops of Levamisol Endogenous Alkaline Phosphatase Inhibitor (Dako, Santa Clara, CA, USA). Then, sections were rinsed with water for 3 minutes and boiled in citrate buffer (pH 6.0, containing 10 mM Tri-sodium Citrate Dihydrate (Merck)) for 10 minutes at 100℃. When cooled down, the sections were incubated with Fluorescin Ulex Europaeus Agglutinin I (Vector Laboratories, diluted 1:40000) for 30 minutes at room temperature, with Ulex Europaeus Lectin Type I Rabbit anti Ulex (Dako, diluted 1:500) for 30 minutes at room temperature and with BrightVision Poly-HRP anti-rabbit IgG (ImmunoLogic, diluted 1:1 with PBS Tween) for 30 minutes at room temperature. HRP activity was visualized using NovaRED (Vector Laboratories).
 for 5 minutes at room temperature in the dark. In all dilutions, Normal Antibody Diluent (ImmunoLogic, Duiven, the Netherlands) was used if not indicated differently. Each incubation was performed at room temperature, lasted 30 minutes and subsequently the sections were rinsed 3 times with PBS-Tween 20 (0,01%) for 3 minutes, unless stated otherwise. Finally, sections were washed in demineralized water and fixed with mounting medium VectaMount (Vector Laboratories, Laboratories, Burlingame, CA, USA) at 60℃.
